# SPECT/CT lymphoscintigraphy of sentinel node(s) for superselective prophylactic irradiation of the neck in cN0 head and neck cancer patients: a prospective phase I feasibility study

**DOI:** 10.1186/1748-717X-9-121

**Published:** 2014-05-28

**Authors:** Jean-François Daisne, Johanne Installé, Benoît Bihin, Marc Laloux, Thierry Vander Borght, Isabelle Mathieu, Georges Lawson

**Affiliations:** 1 Radiation Oncology, Clinique & Maternité Ste-Elisabeth, Place Louise Godin, Namur 15 – 5000, Belgium; 2 Namur Research Institute for Life Sciences (NARILIS), Namur, Belgium; 3 Nuclear Medicine, Clinique & Maternité Sainte-Elisabeth, Namur, Belgium; 4 Unit of Methodology and Didactic in Biology (UMDB), University of Namur, Namur, Belgium; 5 Unit of Biostatistics, CHU Dinant-Godinne, Université Catholique de Louvain, Yvoir, Belgium; 6 Head and Neck Surgery, Clinique & Maternité Sainte-Elisabeth, Namur, Belgium; 7 Nuclear Medicine, CHU Dinant-Godinne, Université Catholique de Louvain, Yvoir, Belgium; 8 Head and Neck Surgery, CHU Dinant-Godinne, Université Catholique de Louvain, Yvoir, Belgium

**Keywords:** Head and neck cancer, Sentinel node, SPECT/CT, Individualized radiotherapy, Target volume selection

## Abstract

**Background:**

Clinically node negative HNSCC patients have a risk ranging between 18 and 45% of occult metastases, making prophylactic irradiation mandatory. Selective irradiation of nodal target volume based on international guidelines is practice. Anyway, about half the tumours lying in an anatomical subsite known to potentially drain bilaterally effectively do so, leading to unnecessary large volume irradiation. Moreover, 15% of the tumours show drainage outside of predicted basin, increasing the risk for potential geographical misses. Three-dimensional SPECT/CT lymphoscintigraphy (LS) of sentinel node(s) may help to individualize nodal target volume selection. This prospective phase I study explores its feasibility and the dosimetric impact.

**Methods:**

Ten cN0 HNSCC patients eligible for definitive radiotherapy were imaged with SPECT/CT after ^99m^Tc nanocolloid injection around the tumour. The neck levels containing up to four hottest nodes were identified and selected for prophylactic irradiation (CTVn-LS) by volumetric modulated arc therapy. A comparative virtual planning was performed with volumes selected according to international guidelines (CTVn-IG).

**Results:**

Migration was observed in all patients (one with gamma probe only). 2.9 sentinel nodes were detected per patient on average. In some patients, accurate localization was difficult when not using thermoplastic mask for SPECT/CT. CTVn-LS was totally encompassed by CTVn-IG in all patients but one (unpredicted drainage in retropharyngeal level). On average, CTVn-LS and related planning target volumes were two times smaller than IG ones. This led to significant dose decrease in identified organs at risk as well as remaining volume at risk.

**Conclusions:**

SPECT/CT LS is a promising tool to individualize prophylactic node CTV in cN0 HNSCC patients eligible for definitive radiotherapy. Oncological safety must be confirmed by ongoing phase II study.

## Background

Surgery and radiotherapy (RT) are the two treatment cornerstones of non-metastatic Head and Neck Squamous Cell Carcinoma (HNSCC). Both modalities have the same treatment philosophy with ultimate goals being cure and restoration of Quality of Life (QoL). These goals are reflected by the therapeutic ratio (i.e. cure rate divided by side effects rate). Both modern surgery and RT aim at improving this ratio by maximizing cure rate and minimizing side effects probability.

In RT, major technical improvements like Three-Dimensional Conformal Radiation Therapy (3D-CRT) and Intensity-Modulated Radiation Therapy (IMRT) have allowed to better conform the dose delivered to the target volumes (TV) while reducing the high doses delivered to Organs at Risk (OAR) and hence, reducing the late toxicity of the treatments compared to treatments with large 2D fields [1]. The accurate selection and definition of the different TV is of utmost importance to maximize the therapeutic ratio when using these highly selective RT techniques. It is particularly true in the head and neck region where lymph drainage is intense with a high incidence of nodal metastases, making irradiation of neck nodes mandatory for most tumours.

The recognition of cancer nodal spread at diagnosis is essential for the prognosis and the extent of nodal surgery or the definition of radiotherapy volumes. Different imaging methods are presently used like Computed Tomography (CT), Ultrasonography (US) without or with Fine Needle Aspiration Cytology (FNAC), and to a lesser extent Magnetic Resonance Imaging (MRI) and/or combined Positron Emission and Computed Tomographies with 18-Fluoro-Deoxy-Glucose (FDG-PET/CT). Their sensitivity and specificity ranges lay roughly between 40 and 100%, being highly dependent on the size of the nodes [2]. Indeed, for nodes showing a diameter smaller or equal to 10 mm in the short axis or showing no central necrosis, no imaging modality is reliable enough to detect tumour deposits, and patients are deemed to be clinically free of nodes (cN0). Anyway, the risk for occult metastasis is still significant, ranging between 18 and 45% in the most recent surgical series exploring the role of sentinel lymph nodes (SLN) mapping in oral cavity, oropharyngeal or laryngo-hypopharyngeal HNSCC [3-13]. These figures preclude cN0 HNSCC patients from a simple watchful waiting attitude. The selection and definition of the predicted drainage nodal basins based on primary tumour location are now well documented by surgeons and pathologists [14] and coded in RT language [15], allowing their (most of the time bilateral) selective treatment by either surgical removal or irradiation at prophylactic dose (typically 45 to 50 Gy by daily fractions of 1.8 to 2.0 Gy).

Though being more selective than older large fields techniques irradiating the whole neck, the current technique still leads to the inclusion of large volumes of potentially normal tissues because 55 to 82% of the cN0 patients are truly free of nodal involvement and bilateral drainage is the rule in only 30 to 50% of individuals presenting a tumour in a region with a known bilateral drainage [3,11,16]. Moreover, it may lead to geographical misses since 15 to 30% of the tumours drain in unpredicted nodal basins [2,5,16,17].

The SLN dissection is a diagnostic surgical procedure based on the injection of a radioactive nanocolloid in the mucosa surrounding the tumour to map the first drainage echelons on an individual basis. The preferential accumulation of the tracer in the SLN allows their per-operative identification with a gamma-probe and their exquisite surgical removal. One to four SLN may be magnified for a given tumour, any other magnified node being no first echelon [3,6,18]. The thorough pathological analysis of these nodes predicts the nodal stage of the neck with reported sensitivity, specificity and negative predictive values ranging between 73–100%, 78–100% and 83–100%, respectively [3,4,6,7,9-11,17,19]. The sensitivity of the method is highest when a superselective nodal dissection is performed (i.e. removal of the level where the SLN is located) [3,5] and when performed by an experienced team [9].

This SLN mapping concept is appealing for the head and neck radiation oncologist because it may help to individually tailor the nodal prophylactic irradiation volume in cN0 patients. Anyway, current surgical technique of SLN localization with planar lymphoscintigraphy and per-operative gamma-probe is not accurate enough for radiotherapy planning. The recent development of a 3D Lymphoscintigraphy (LS) based on Single Photon Emission Computed Tomography coupled with a Computed Tomography (SPECT/CT) allows acquiring composite images that could overcome this limitation by making possible the accurate localization of the SLN [8,17,20,21].

The transfer of this diagnostic surgical method to the technical context of radiotherapy, where no pathological analysis is possible, first needs to be studied according to a robust methodology. Indeed, key questions regarding both the feasibility and the true reduction of treatment volumes without increasing the risk of metachronous relapses need to be answered before being tested in the frame of a phase III randomized study.

A prospective phase I/II study was designed to test the feasibility and the oncologic safety of the SLN mapping method applied to the tailored prophylactic irradiation of nodal stations in HNSCC patients being clinically N0. We report here phase I results aimed at establishing the feasibility and limitations of the SPECT/CT lymphoscintigraphy integration in the frame of RT specificities. Ten patients were included and treated superselectively according to individual data. A comparative planning with selective prophylactic irradiation according to international guidelines (IG) [15] was made for all patients to measure potential dose reduction to normal tissues.

## Methods

### Patients and enrolment

Ten patients with non-operated pathologically proven invasive HNSCC were prospectively invited to participate. Eligibility criteria included: age ≥ 18 years; referred by oncology multidisciplinary team for primary radiotherapy (with or without sensitization); no neo-adjuvant chemotherapy or surgery; World Health Organization performance status 0 or 1; any cT-stage primary tumour located in the oral cavity, oropharynx, larynx (excepted tumours strictly localized to the glottic plane) or hypopharynx presenting a risk of nodal spread justifying a prophylactic node irradiation; no macroscopic nodal or distant metastasis spread as assessed by FDG-PET/CT and CT contrast agent injection. Nodes were deemed cN0 if shortest diameter was ≤ 5 mm at retropharyngeal level and ≤ 10 mm at any other level and/or exhibiting no central necrosis. In dubious cases (high FDG uptake or size in level 2 between 11 and 15 mm), a US with FNAC had to be performed. Before registration, written informed consent had to be given according to ICH/GCP, and national/local regulations. Quality of Life (QoL) assessment was performed the same day using EORTC C30 and HN25 scales. Exclusion criteria were: pregnancy or no active contraception for non-menopausal women; HNSCC originating from nose, sinuses, oesophagus, salivary glands or nasopharynx; non-HNSCC histology; second malignancy; previous history of cancer in the last 5 years (excluding basal cell carcinoma of the skin and in situ SCC of the cervix); known hypersensitivity to iodine or nanocolloid injection; violated neck (i.e. previous surgery or radiotherapy to the neck); any psychological, familial, sociological or geographical condition potentially hampering compliance with the study protocol and follow-up schedule. The study protocol and informed consent form were reviewed and approved by the Ethics Committees of CHU Dinant-Godinne and Clinique & Maternité Sainte-Elisabeth on November 8^th^, 2012 (National Belgian Reference Number: B039201215085).

### Nanocolloid injection and SPECT/CT lymphoscintigraphy

Patients were simulated with their head blocked in a five points thermoplastic mask according to our standard protocol [22]. Within the next seven days, patients were referred for SPECT/CT lymphoscintigraphy. Patients with oral cavity and/or accessible oropharyngeal tumours were injected without sedation in the Nuclear Medicine department, the others during an endoscopy under a short sedation in operating room [11]. Because surgical expertise is of utmost importance [9], only two surgeons having extensive experience with the SLN technique performed all injections (ML for oropharyngeal and oral cavity tumours; GL for laryngeal and hypopharyngeal tumours). ^99m^Tc labelled human serum albumin colloid (Nanocoll®, GE Healthcare, Diegem, Belgium) was used as radioactive tracer (18,5-37 MBq in 1 ml) and injected submucosally in four aliquots at one to five millimetres from macroscopic tumour edges, according to standard protocol used for surgical procedures [3,11]. Nanocoll® migration in SLN was verified with dynamic planar lymphoscintigraphy for patients without sedation or in the operating room with a hand-held kneed gamma probe (Navigator^TM^, RMD Instruments, LLC, Watertown, MA, USA) before transfer to Nuclear Medicine department when sedated.

SPECT/CT images were acquired on hybrid cameras, either a Siemens SYMBIA T (Siemens, Erlangen, Germany) or GE Discovery NM/CT 670 (GE Healthcare, Waukesha, WI, USA) camera in uncontrolled position for the first seven patients and for the next ones on a carbon tabletop with RT mask positioned. SPECT acquisition parameters were: 128 × 128 matrix, zoom 1, 2 detectors at 180°, 64 steps of 20 sec with the Siemens device or 60 steps of 15 sec for the GE one. Low-dose CT acquisition parameters for the Siemens or the GE camera were respectively: slice thickness 1.25 or 0.625 mm, 130 or 120 kV and dose modulation with « Care Dose 4D » or modulation between 50 and 380 mAs. SPECT data were iteratively reconstructed using CT data for attenuation correction.

Reconstructed images were transferred to a Mac Pro under MacOSX 10.8.3 (Apple, Palo Alto, CA, USA) running the OsiriX MD v2.0.1 software (Pixmeo, Bernex, Switzerland). Both the radiation oncologist and the nuclear medicine specialist visually inspected fused images for identification of all SLN that were recorded in descending order of relative maximal activity and anatomical localization according to standardized RT nomenclature [15]. Only the four hottest nodes were considered for radiotherapy planning; for patients exhibiting more than four SLN, they were recorded as well but not considered for planning.

### Radiotherapy planning

Primary Gross Tumour Volume (GTVp) was delineated according to our current practice on Eclipse Treatment Planning System (Varian, Palo Alto, USA): CT-based, modulated by clinical examination and MRI if available, a five to 10 mm anisotropic margin being added to generate the primary Clinical Target Volume (CTVp). Defined OAR were: spinal cord, brainstem, parotids, submandibular glands, swallowing muscles [23], oral cavity for larynx/hypopharynx tumours, larynx for oral/oropharyngeal tumours and Remaining Volume at Risk (RVR) after final Planning Target Volume (PTV) delineation (RVR = body – PTV_total_ – all OAR). Two different prophylactic nodes Clinical Target Volume (CTVn) were defined: one “superselective” for treatment purpose (real treatment plan) based on lymphoscintigraphy findings (CTVn-LS) selecting all node levels containing the four hottest SLN [3,6,18]. The whole level rather than the SLN alone had to be selected because most of positive non-SLN nodes are located in the same level [5]. Another one for comparison purpose (virtual treatment plan) was planned based on international guidelines (CTVn-IG) with node levels selected according to primary tumour location and extension (“selective”). For putative patient(s) exhibiting no SLN, CTV considered for treatment would have been the IG one. Various CTVs were expanded by 4 mm to obtain the related PTVs.

Altered fractionation was prescribed if radiotherapy alone was used (PTVp = 69 Gy in 30 fractions of 2.3 Gy; PTVn = 55.5 Gy in 30 fractions of 1.85 Gy); conventional fractionation was prescribed if sensitizing cisplatinum or cetuximab had to be used (PTVp = 70 Gy in 35 fractions of 2.0 Gy; PTVn = 59.5 Gy in 35 fractions of 1.7 Gy).

Planning was performed with a Simultaneous Integrated Boost technique (SIB) using volumetric arc modulated radiotherapy (RapidArc, Varian, Palo Alto, CA, USA), dose calculations being done with Analytical Anisotropic Algorithm (AAA, Varian, Palo Alto, CA, USA). PTV coverage had to comply with ICRU 83 recommendations [24] while keeping D_2%_ to spinal cord and brainstem below 45 and 50 Gy, respectively. Maximal optimization was required on all defined OAR. RVR dose was constrained through the use of concentric rings around the PTV. To avoid any bias, the same dosimetrist optimized both plans at the same time, always starting with the LS-one.

### Statistics

R (version 2.15.1, The R Foundation for Statistical Computing, Vienna, Austria) was used for statistical analysis. As normality could not be assumed, median and interquartile range were computed and differences between planning methods were analyzed with Wilcoxon signed rank test with continuity correction. *P*-values less than .05 were considered statistically significant.

## Results

### Patients

Ten patients fulfilling inclusion criteria accepted to participate and signed informed consent between 9 January and 24 June 2013. There were nine males and one female; age ranged from 43 to 89 years (mean 63). Tumours locations and cT-stages: seven in the larynx (three T2 and four T3, all with supra- and/or sub-glottic extension and hence, increased risk of nodal dissemination); two in the oropharynx (both T2) and one in the oral cavity (small T4 of the hard palate mucosa infiltrating the underlying bone). All tumors were judged eligible by the surgeons for peri-tumoral nanocolloid injection. Seven patients were treated by altered fractionation, three by conventional fractionation (one with concomitant three-weekly cisplatinum and two with weekly cetuximab). All were treated according to LS volumes. All ended their radiotherapy course within the prescribed overall treatment time.

### Nanocolloid migration and target volume selection

**Figure 1:**
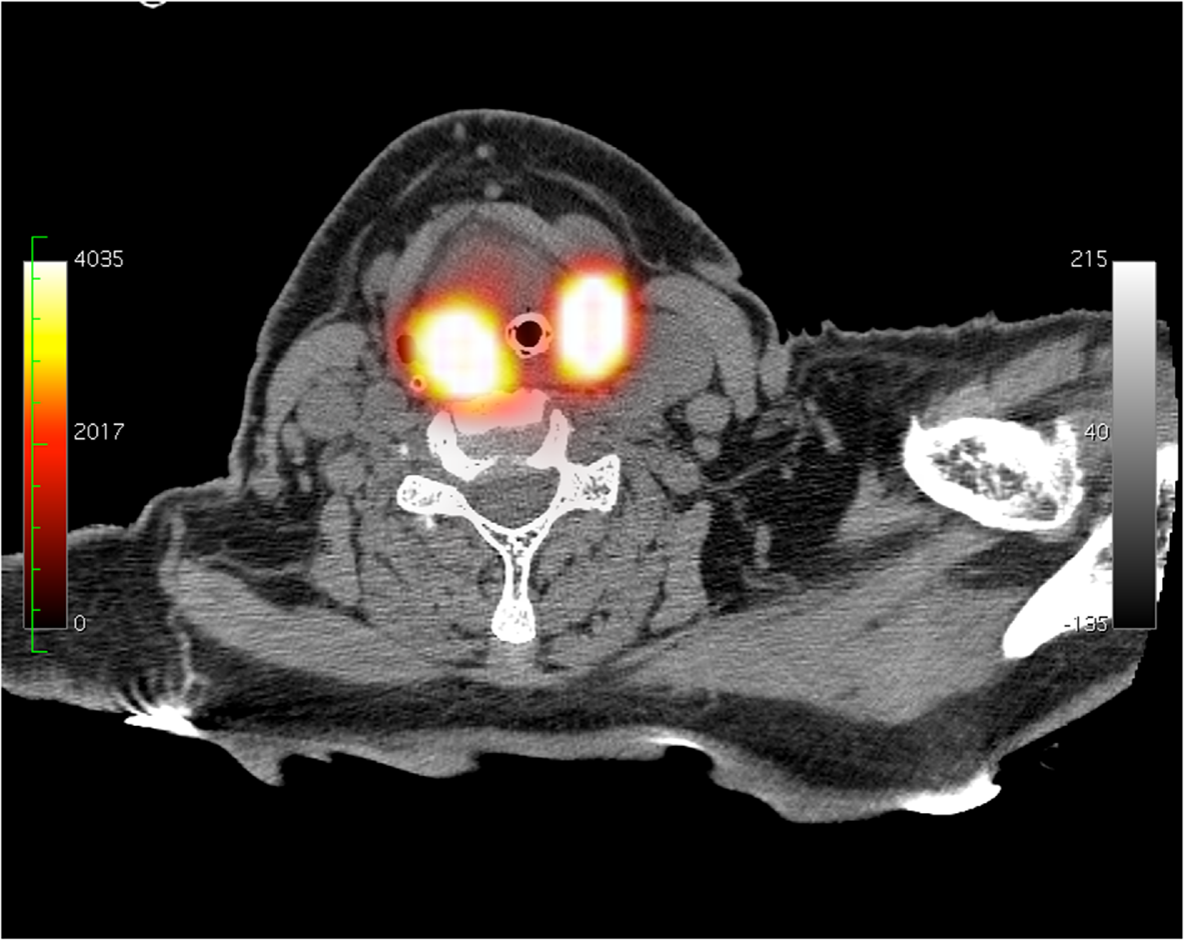
**Axial SPECT/CT slice, patient 6.** No apparent migration is depicted in level 3 left, probably due to intense activity in neighbouring tumour.

**Figure 2:**
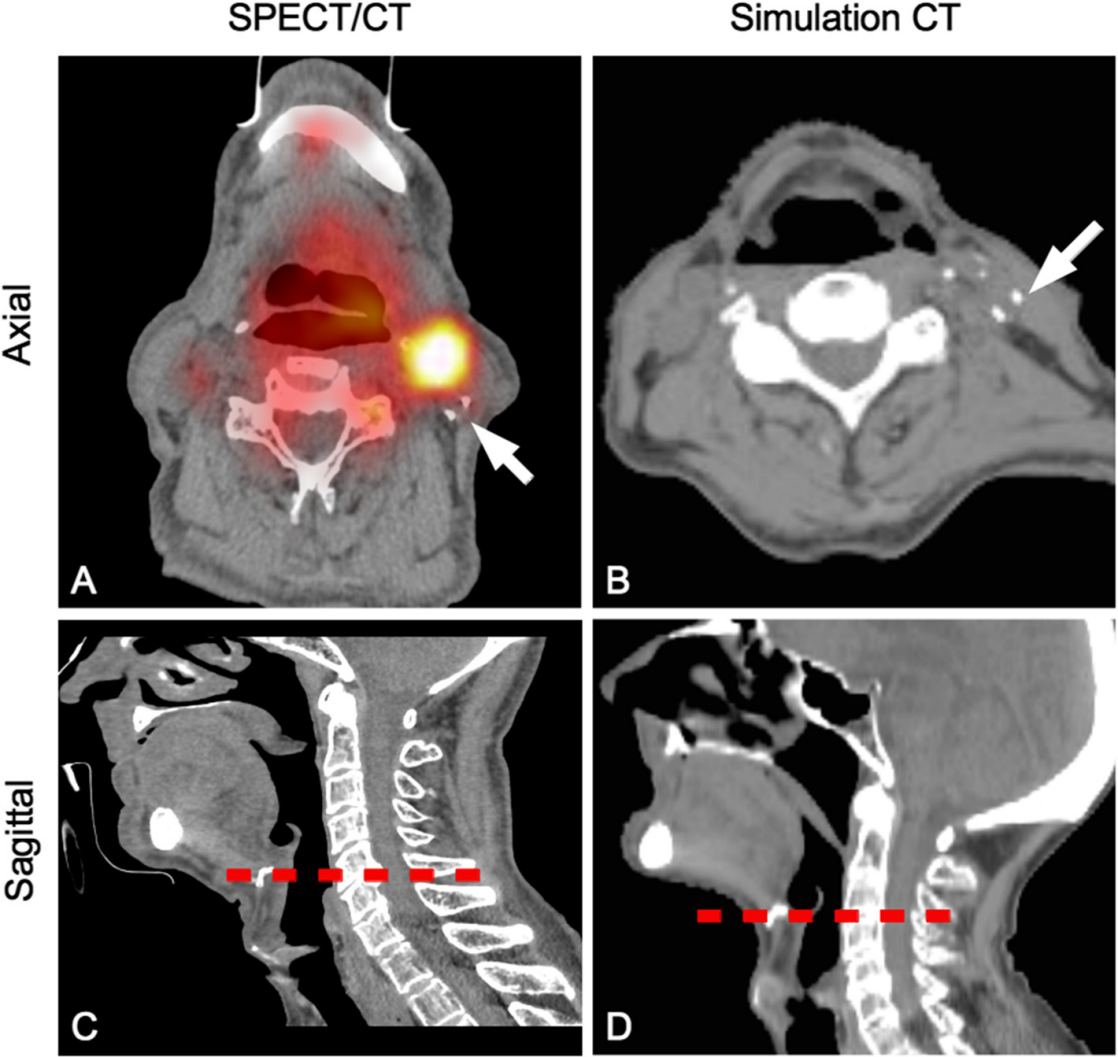
**Inaccuracy in node level localization due to variations in head position.** For patient 7, the correct localization of the sentinel node on simulation CT **(B)** was based on vascular calcifications on SPECT/CT **(A)** (white arrows). The discordance between SPECT/CT (level 2) and simulation CT (level 3) is due to uncontrolled position of the head for SPECT/CT acquisition and subsequent relative position of hyoid bone (dotted red line, **C** and **D**).

Nanocoll® migration was observed in all patients either on planar LS or with hand-held gamma probe. SPECT/CT migration was observed in all patients except patient 6 (larynx, T3 – Figure 1) where tumour burden hid the SLN activity, detected subsequently by gamma probe in the homolateral level 3. On average, 2.9 nodes were detected per patient: one in two patients, two in three patients, three in two patients, four in one patient, five in one patient and six in one patient (Table 1). CTVn-LS volumes were selected based on the four hottest nodes since any supplementary highlighted node is not considered as a first echelon [3,6,18]. For patients 4 and 7 the localization on the planning CT was difficult because of patient position differences between SPECT/CT and planning CT (Figure 2). From patient 8 onwards, SPECT/CT were acquired with patient lying on a flat carbon couch and immobilized with its radiotherapy thermoplastic mask to allow easier accurate SLN localizations.

**Table 1:** Tumour details per patient

**Patient #**	**1**	**2**	**3**	**4**	**5**	**6**	**7**	**8**	**9**	**10**	**Median**
**Localization**	**Oropharynx**	**Larynx**	**Oral cavity**	**Larynx**	**Oropharynx**	**Larynx**	**Larynx**	**Larynx**	**Larynx**	**Larynx**	
Laterality	L	L	L	L	L	B	B	R	R	B	
cT-stage	2	3	4	3	2	3	2	2	3	2	
Number of SLN	4	3	1	5	2	1	2	3	2	6	
CTVn-LS selection											
Levels left	2, 3	2, 3	-	3, 6	2	3	3	-	-	3	
Levels right	RS	-	2	3, 6	2	-	3	2-4	2-4	3,4	
Volume (cc)	141.8	80.3	119.8	144.2	74.8	87.8	67.5	88.4	101.2	96.7	92.6
Volume PTVn-LS (cc)	287.5	191.5	267.6	277.2	204.7	196.8	198.9	216.1	245.8	243.5	229.8
CTVn-IG selection											
Levels left	2 - 4	2 - 4	1b - 4	2-4, 6	2-4	2-4	2-4	2-4	2-4	2-4, 6	
Levels right	2 - 4	2 - 4	1b - 4	2-4, 6	2-4	2-4	2-4	2-4	2-4	2-4, 6	
Volume (cc)	223.0	160.8	225.0	219.3	154.8	215.7	118.7	195.3	179.6	209.0	202.2
Volume PTVn-IG (cc)	451.4	405.3	508.4	447.0	404.1	465.6	330.2	476.6	428.7	492.2	449.2

SLN = sentinel lymph node; L = left; R = right; B = bilateral; RS = retrostyloid.

Volumes selection and measurements are based either on lymphoscintigraphy (LS) or international guidelines (IG). Median volumes are reported in last column.

**Figure 3:**
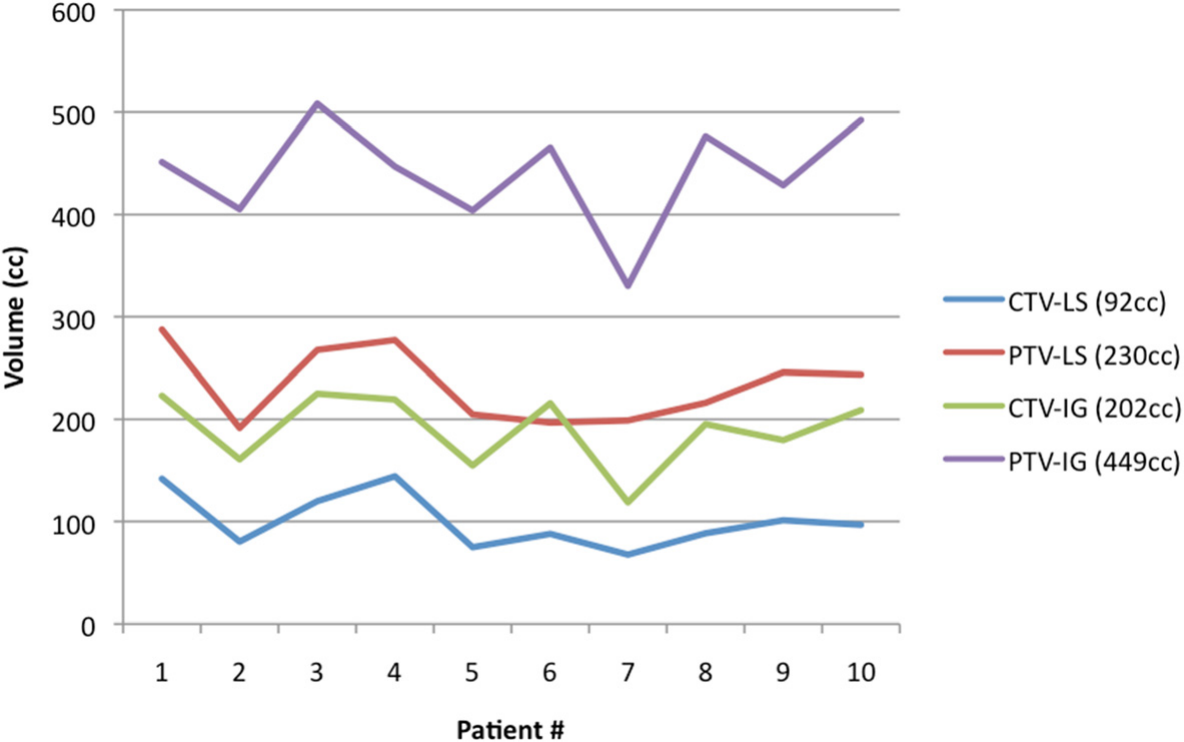
**Measured target volumes for the different patients, based on international guidelines (IG) or lymphoscintigraphy (LS).** Respective median values are written between parentheses.

**Figure 4:**
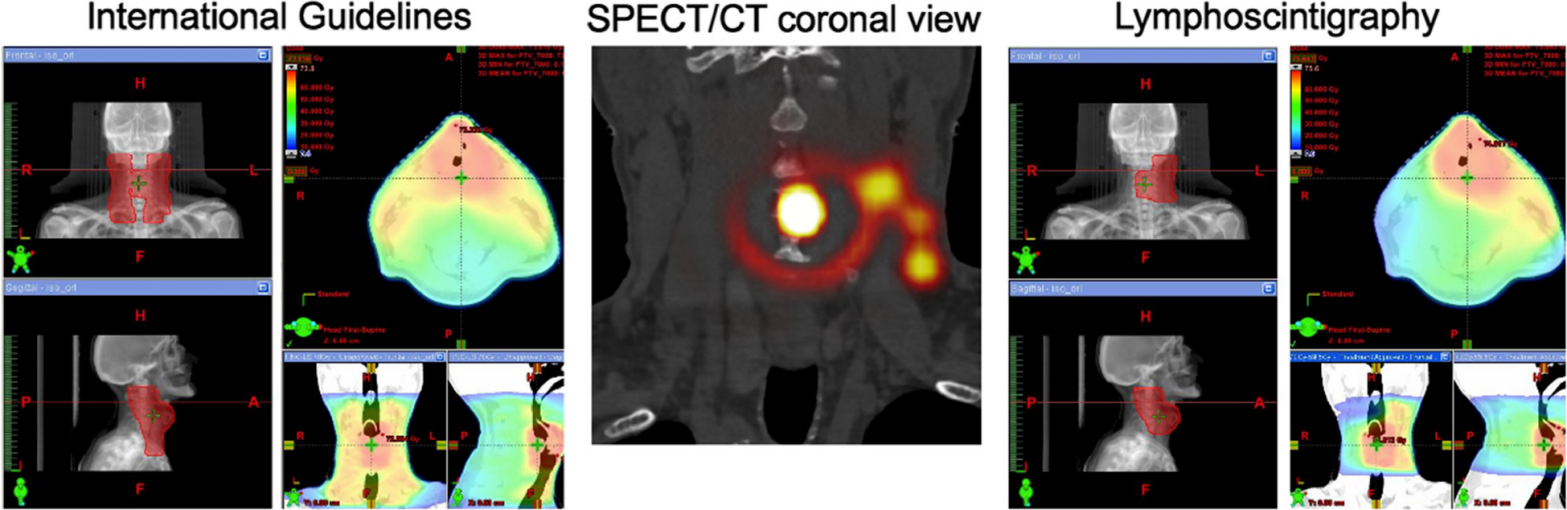
**Impact of lymphoscintigraphy on volumes selection and dose distribution for patient 2.** Left: PTV based on international guidelines projected on Digitally Reconstructed Radiographies (DRR) and correlated 3D dose distribution. Centre: SPECT/CT coronal view depicting 1 node caudally in level 2 left and 2 other ones in level 3 left. Right: PTV selection based on lymphoscintigraphy projected on DRR and correlated 3D dose distribution.

The CTVn-LS volume was totally encompassed by CTVn-IG volumes except in one case (patient 1) where migration in homolateral retrostyloid region was unpredicted by IG (Table 1). LS volumes were systematically smaller than related IG ones, by a factor of two on average (*P* = 0.006) (Figure 3). As example, the differences in CTVn selections are depicted for patient 2 in Figure 4.

### Dosimetry

CTVn-LS and CTVn-IG were correctly covered in all patients with lower D_98%_ of 96.6 and 96.5%, respectively and lower D_95%_ of 98.9 and 97.8%, respectively. The same holds true for the PTVs except for patient 4 due to build-up effect in the level 6 where the PTV expanded in the air anteriorly (Table 2).

**Table 2:** Target volumes coverage for plans based on either lymphoscintigraphy (LS) volumes or international guidelines (IG) ones

**Patient #**	**1**	**2**	**3**	**4**	**5**	**6**	**7**	**8**	**9**	**10**	**Median**	***P*-value**
CTVn-LS doses (%)												
D_95%_	100.2	103.7	100.0	99.2	100.9	102.9	99.8	98.9	99.8	99.5	99.91	0.232
D_98%_	98.7	102.2	98.9	96.6	99.8	101.7	99.3	98.4	99.3	98.6	99.10	0.284
D_mean_	111.4	113.9	117.7	107.1	118.7	115.0	107.4	108.5	107.2	108.3	109.91	0.006
PTVn-LS doses (%)												
D_95%_	98.7	100.5	98.4	90.9	100.4	101.5	98.7	98.7	99.1	98.9	98.83	0.059
D_98%_	97.5	97.5	88.5	80.0	99.1	100.2	96.9	98.2	98.4	98.2	97.84	0.185
D_mean_	111.0	111.8	114.2	102.9	117.1	114.1	106.1	108.5	106.3	106.8	109.73	0.006
CTVn-IG doses (%)												
D_95%_	98.2	99.5	100.0	97.8	100.9	101.2	100.5	99.6	99.3	100.2	99.82	-
D_98%_	96.6	98.3	99.3	96.5	99.3	100.2	99.8	98.7	98.6	99.5	99.01	-
D_mean_	110.1	107.1	110.8	105.5	112.4	109.6	106.3	105.8	104.7	106.5	106.77	-
PTVn-IG doses (%)												
D_95%_	96.6	96.5	98.7	90.4	98.9	99.2	98.6	98.9	98.7	99.1	98.74	-
D_98%_	88.1	94.1	95.1	82.0	96.6	97.3	95.5	98.0	98.0	97.3	96.04	-
D_mean_	109.2	105.2	108.6	101.8	111.0	108.4	105.2	105.4	104.3	104.7	105.32	-

CTVn = prophylactic nodes clinical target volume; PTVn = prophylactic nodes planning target volume; D_95%_ = dose received by 95% of the volume; D_98%_ = dose received by 98% of the volume; D_mean_ = mean dose.

*P*-values for statistical differences for LS values compared to IG ones are reported in last column; level of significance is set at 0.05.

Doses delivered to OAR were significantly reduced in LS plans, except for the spinal cord and the low pharyngeal constrictor muscle (LPC) (Table 3). Regarding the spinal cord, the integral dose was in all cases reduced. The absence of statistical difference for LPC is related to primary tumours locations, being the larynx for seven patients.

**Table 3:** Comparative dosimetry after planning based on lymphoscintigraphy (LS) or international guidelines (IG) volumes

	**LS**	**IG**	***P*-value**
**Nervous structures**			
Spinal cord D_2%_ (Gy)	41.7	43.4	0.083
Brainstem D_2%_ (Gy)	4.2	23.7	**0.013**
**Salivary glands**			
Left parotid D_mean_ (Gy)	4.2	25.0	**0.006**
Right parotid D_mean_ (Gy)	10.9	25.4	**0.006**
Left submand D_mean_ (Gy)	22.6	46.9	**0.009**
Right submand D_mean_ (Gy)	27.4	42.7	**0.014**
**Swallowing muscles**			
SPC D_mean_ (Gy)	22.3	41.2	**0.009**
MPC D_mean_ (Gy)	45.1	54.6	**0.041**
LPC D_mean_ (Gy)	55.4	56.4	0.154
UES D_mean_ (Gy)	39.8	54.3	**0.006**
Oesophagus D_mean_ (Gy)	9.6	43.6	**0.006**
**Oral cavity Dmean (Gy)**	15.0	20.3	**0.024**
**RVR volume at D**_**proph**_**(cc)**	63.7	108.4	**0.006**

D_2%_ = dose received by 2% of the volume; D_mean_ = mean dose; Submand = submandibular gland; SPC = superior pharyngeal constrictor muscle; MPC = middle pharyngeal constrictor muscle; LPC = low pharyngeal constrictor muscle; UES = upper oesophageal sphincter.

Median D_2%_ for nervous structures; median D_mean_ for salivary glands, swallowing structures and oral cavity and median volume of remaining volume at risk (RVR) treated at prophylactic dose (D_proph_) are reported. *P*-values for statistical comparison of differences are reported in last column; significant values are written in bold.

## Discussion

In this prospective phase I study, we demonstrate the potential of SPECT/CT-based LS of SLN for non-operated cN0 HNSCC patients eligible for definitive irradiation. It allows individualizing the superselective selection of prophylactic nodal target volume while significantly reducing normal tissues irradiation. Two potential pitfalls were also highlighted. The first concerns small inaccuracies in SLN localization when patient position is not controlled, which can be overcome by acquiring SPECT/CT with radiotherapy thermoplastic mask on a flat carbon couch. The second relates to non-migration of colloid into SLN. The incidence is low, ranging from 0% [6,11] to 7% [10]. In this case, we recommend selecting target volume based on IG rather than avoiding prophylactic nodal irradiation since it may be due either to node hilus blockage by tumour cells or delay in migration justifying late acquisition on SPECT/CT [6,20]. Rarely, the SLN that may be hidden on SPECT/CT images by the intense activity around the tumour may be detected by the unidirectional handheld gamma probe [17]. This is also documented in floor of mouth tumours where gamma probe may even be inefficient [2,10]. It happened for patient 6 where SPECT-CT did not image any SLN while the unidirectional handheld probe could detect one in level 3, in the close vicinity of the larynx tumour.

The integration of SPECT/CT LS information to radiotherapy plans is quite recent with two papers reporting its potential interest for prostate [25] and breast [26] cancers treatment. In a prospective series of 20 prostate cancer patients with a nodal risk ≥ 20%, SPECT/CT acquired 90 minutes after injection of nanocolloid in prostate magnified 27 SLN outside of standard CTV in 14 patients. After exclusion of paraaortic and inguinal nodes, there were still nine pararectal nodes magnified leading to a modification of CTV in six patients (30%) [25]. In breast cancer, standard tangential fields imply unnecessary irradiation of arm draining lymph nodes, increasing the risk for lymphedema. A prospective study on 28 patients aimed at identifying these nodes with SPECT/CT after injection of nanocolloid in the arm. The modification of standard fields to block the 57% of nodes included in the standard fields, led to a reduction of mean dose to these nodes from 23.6 to 7.7 Gy, without compromising target irradiation. No patient developed lymphedema [26].

In the head and neck area, this is the first time that SPECT/CT LS is used to individualize TV selection. Nevertheless, some key questions still must be answered, oncological safety being the most important. Patient recruitment is maintained through prolongation in a phase II study (44 patients in total) aiming at establishing the true risk of relapse outside of TV at two years. In surgical series, skip metastases (i.e. metastases in non SLN) or metachronous nodal relapse rates are on average of the order of 5% (range [3–10%]) [3,6,12,13]. In surgical cases, a positive SLN leads to complimentary neck dissection. Additional infiltrated non-SLN nodes are mainly located in the same level [5]. This is the safety reason why we decided to include the full level(s) in the CTVn rather than the SLN alone. The risk for occult nodal metastases outside of involved levels is 7% of all positive cases, which sets the global risk at less than 2% of all patients [5]. This risk is sufficiently negligible to avoid any further enlargement of the CTVn. Another key question relates to the limitation of the highlighted nodes to four since any supplementary node is not considered as first echelon [3,6,18]. Further follow-up will learn us if potentially relapsing patients will do so in highlighted nodes not included in the CTVn-LS.

A randomized phase III study could be initiated if nodal relapse outside of TV is < 10%. Other key questions like impact on overall survival and quality of life should also be answered in the future to confirm the potential added value of this technique.

## Conclusions

In cN0 HNSCC patients, individualized superselective prophylactic irradiation of nodal TV was investigated with use of SLN SPECT/CT lymphoscintigraphy information. This phase I study on 10 patients demonstrated the feasibility and the possible pitfalls of the technique. Compared to selective CTVn selection according to international guidelines, the PTV was reduced by a factor of two. Using VMAT planning, a significant decrease in normal tissues irradiation could be achieved. The safety must be confirmed by the ongoing phase II study.

## Abbreviations

2D: Two-dimensional; 3D: Three-dimensional; 3D-CRT: Three-dimensional conformal radiation therapy; AAA: Analytical anisotropic algorithm; cN0: Clinically free of nodes; CT: Computed tomography; CTVn: Prophylactic nodes clinical target volume; CTVp: Primary clinical target volume; Dx%: Dose received by x% of the volume; Dmean: Mean dose; Dproph: Prophylactic dose; DRR: Digitally reconstructed radiographies; FDG-PET/CT: Combined Positron emission and computed tomographies with 18-fluoro-deoxy-glucose; FNAC: Fine needle aspiration cytology; GTVp: Primary gross tumour volume; HNSCC: Head & neck squamous cell carcinoma; IG: International guidelines; IMRT: Intensity-modulated radiation therapy; L: Left; LPC: Low pharyngeal constrictor muscle; LS: Lymphoscintigraphy; MPC: Middle pharyngeal constrictor muscle; MRI: Magnetic resonance imaging; OAR: Organs at risk; PTV: Planning target volume; PTVn: Prophylactic nodes planning target volume; PTVp: Primary planning target volume; QoL: Quality of life; R: Right; RS: Retrostyloid; RT: Radiotherapy; RVR: Remaining volume at risk; SIB: Simultaneously integrated boost; SLN: Sentinel lymph node; SPC: Superior pharyngeal constrictor muscle; SPECT/CT: Single photon emission computed tomography coupled with a computed tomography; Submand: Submandibular gland; TV: Target volume; UES: Upper oesophageal sphincter; US: Ultrasonography.

## Competing interests

All authors have no financial or non-financial competing interests to declare.

## Authors’ contributions

JFD conceived the study, elaborated the design, acquired and analyzed clinical data and drafted the manuscript. JI elaborated the scintigraphic methodology and participated to the analysis of the SPECT/CT data. BB performed the statistical analysis. ML performed nanocolloid injections around oropharyngeal and oral cavity tumours. TVB and IM participated to the analysis of the SPECT/CT data. GL elaborated the sentinel lymph node injection methodology and performed nanocolloid injections around laryngeal tumours. All authors read and approved the final manuscript.

## Authors’ information

JFD was PhD fellow (1999 – 2002) and clinical fellow (2002–2003 and 2005–2006) of Professor Vincent Grégoire in Université Catholique de Louvain. He performed his PhD thesis on multimodality imaging for head and neck cancer radiotherapy treatment. He currently is Head of Radiation Oncology Dept at Clinique & Maternité Ste-Elisabeth in Namur. 40% of his activity relates to HNC patients treatment, with on average two new HNC patients a week; 20% of activity is devoted to imaging and radiation oncology translational research in the frame of the academic structure NAmur Research Institute for Life Sciences (NARILIS).

JI and IM are Nuclear Medicine physicians since more than 10 years, with a daily practice of PET/CT and SPECT/CT imaging. They also have expertise in sentinel node imaging of breast, melanoma, gynaecological and head & neck cancers.

ML is Head & Neck surgeon with expertise in the cancer surgery and the reconstruction surgery.

BB is research assistant in biostatistics at both University of Namur and CHU Dinant-Godinne.

TVB is Head of the Nuclear Medicine Dept in CHU Dinant-Godinne. He co-authored with GL papers and posters on sentinel node imaging in the Head & Neck.

GL is ENT, Head & Neck surgeon and Head of Surgical Dept, University Hospital CHU Dinant-Godinne (Université Catholique de Louvain). He has a long surgical experience of sentinel node in Head & Neck Cancer and published many papers on the subject. He is a member of the steering committee of the European research group on Sentinel node in Head & Neck Cancer; the NAmur Research Institute for Life Sciences (NARILIS) and the Institut de Recherche Expérimentale et Clinique (Université catholique de Louvain – IREC).

## References

[B1] Nutting CM Morden JP Harrington KJ Urbano TG Bhide SA Clark C Miles EA Miah AB Newbold K Tanay M Adab F Jefferies SJ Scrase C Yap BK A’Hern RP Sydenham MA Emson M Hall E PARSPORT Trial Management Group Parotid-sparing intensity modulated versus conventional radiotherapy in head and neck cancer (PARSPORT): a phase 3 multicentre randomised controlled trial Lancet Oncol 2011 12 127 136 10.1016/S1470-2045(10)70290-4 21236730 PMC3033533

[B2] Coughlin A Resto VA Oral cavity squamous cell carcinoma and the clinically n0 neck: the past, present, and future of sentinel lymph node biopsy Curr Oncol Rep 2010 12 129 135 10.1007/s11912-010-0090-7 20425598 PMC2862587

[B3] Werner JA Dünne AA Ramaswamy A Dalchow C Behr T Moll R Folz BJ Davis RK The sentinel node concept in head and neck cancer: solution for the controversies in the N0 neck? Head Neck 2004 26 603 611 10.1002/hed.20062 15229903

[B4] Höft S Maune S Muhle C Brenner W Czech N Kampen W-U Jänig U Laudien M Gottschlich S Ambrosch P Sentinel lymph-node biopsy in head and neck cancer Br J Cancer 2004 91 124 128 10.1038/sj.bjc.6601877 15188012 PMC2364744

[B5] Stoeckli SJ Sentinel node biopsy for oral and oropharyngeal squamous cell carcinoma of the head and neck Laryngoscope 2007 117 1539 1551 10.1097/MLG.0b013e318093ee67 17667135

[B6] Tomifuji M Shiotani A Fujii H Araki K Saito K Inagaki K Mukai M Kitagawa Y Ogawa K Sentinel node concept in clinically n0 laryngeal and hypopharyngeal cancer Ann Surg Oncol 2008 15 2568 2575 10.1245/s10434-008-0008-x 18574637

[B7] Santaolalla F Sanchez JM Ereño C Gonzalez A Rodriguez ML Sanchez A Martinez A Non-sentinel node tumor invasion in oropharyngeal and oral cancer: risk of misdiagnosis of metastasis Acta Otolaryngol 2008 128 1159 1164 10.1080/00016480801891710 18607950

[B8] Stephan KH Sandro JS SPECT/CT for lymphatic mapping of sentinel nodes in early squamous cell carcinoma of the oral cavity and oropharynx Int J Mol Imaging 2011 2011 106068 21490726 10.1155/2011/106068 PMC3065910

[B9] Civantos FJ Zitsch RP Schuller DE Agrawal A Smith RB Nason R Petruzelli G Gourin CG Wong RJ Ferris RL Naggar El A Ridge JA Paniello RC Owzar K McCall L Chepeha DB Yarbrough WG Myers JN Sentinel lymph node biopsy accurately stages the regional lymph nodes for T1-T2 oral squamous cell carcinomas: results of a prospective multi-institutional trial J Clin Oncol 2010 28 1395 1400 10.1200/JCO.2008.20.8777 20142602 PMC2834497

[B10] Alkureishi LWT Ross GL Shoaib T Soutar DS Robertson AG Thompson R Hunter KD Sorensen JA Thomsen J Krogdahl A Alvarez J Barbier L Santamaria J Poli T Sesenna E Kovács AF Grünwald F Barzan L Sulfaro S Alberti F Sentinel node biopsy in head and neck squamous cell cancer: 5-year follow-up of a European multicenter trial Ann Surg Oncol 2010 17 2459 2464 10.1245/s10434-010-1111-3 20552410

[B11] Lawson G Matar N Nollevaux M-C Jamart J Krug B Delos M Remacle M Vander Borght T Reliability of sentinel node technique in the treatment of N0 supraglottic laryngeal cancer Laryngoscope 2010 120 2213 2217 10.1002/lary.21131 20949579

[B12] Yoshimoto S Hasegawa Y Matsuzuka T Shiotani A Takahashi K Kohno N Yoshida T Kitano H Sentinel node biopsy for oral and laryngopharyngeal squamous cell carcinoma: a retrospective study of 177 patients in Japan Auris Nasus Larynx 2012 39 65 70 10.1016/j.anl.2011.03.002 21592700

[B13] Gurney BAS Schilling C Putcha V Alkureishi LW Alvarez AJ Bakholdt V Barbier Herrero L Barzan L Bilde A Bloemena E Salces CC Dalla Palma P de Bree R Dequanter D Dolivet G Donner D Flach GB Fresno M Grandi C Haerle S Huber GF Hunter K Lawson G Leroux A Lothaire PH Mamelle G Silini EM Mastronicola R Odell EW O’Doherty MJ Implications of a positive sentinel node in oral squamous cell carcinoma Head Neck 2012 34 1580 1585 10.1002/hed.21973 22290737

[B14] Werner JA Dünne AA Myers JN Functional anatomy of the lymphatic drainage system of the upper aerodigestive tract and its role in metastasis of squamous cell carcinoma Head Neck 2003 25 322 332 10.1002/hed.10257 12658737

[B15] Grégoire V Levendag P Ang KK Bernier J Braaksma M Budach V Chao C Coche E Cooper JS Cosnard G Eisbruch A El-Sayed S Emami B Grau C Hamoir M Lee N Maingon P Muller K Reychler H CT-based delineation of lymph node levels and related CTVs in the node-negative neck: DAHANCA, EORTC, GORTEC, NCIC, RTOG consensus guidelines Radiother Oncol 2003 69 227 236 10.1016/j.radonc.2003.09.011 14644481

[B16] Wagner A Schicho K Glaser C Zettinig G Yerit K Lang S Klug C Leitha T SPECT-CT for topographic mapping of sentinel lymph nodes prior to gamma probe-guided biopsy in head and neck squamous cell carcinoma J Craniomaxillofac Surg 2004 32 343 349 15555515 10.1016/j.jcms.2004.05.008

[B17] Haerle SK Hany TF Strobel K Sidler D Stoeckli SJ Is there an additional value of SPECT/CT over planar lymphoscintigraphy for sentinel node mapping in oral/oropharyngeal squamous cell carcinoma? Ann Surg Oncol 2009 16 3118 3124 10.1245/s10434-009-0632-0 19636629

[B18] Atula T Shoaib T Ross GL Gray HW Soutar DS How many sentinel nodes should be harvested in oral squamous cell carcinoma? Eur Arch Otorhinolaryngol 2008 265 Suppl 1 S19 S23 18092173 10.1007/s00405-007-0548-x PMC2441492

[B19] Christensen A Bilde A Therkildsen MH Mortensen J Charabi B Kirkegaard J Specht L Buchwald CV The prevalence of occult metastases in nonsentinel lymph nodes after step-serial sectioning and immunohistochemistry in cN0 oral squamous cell carcinoma Laryngoscope 2011 121 294 298 10.1002/lary.21375 21271576

[B20] Khafif A Schneebaum S Fliss DM Lerman H Metser U Ben-Yosef R Gil Z Reider-Trejo L Genadi L Even-Sapir E Lymphoscintigraphy for sentinel node mapping using a hybrid single photon emission CT (SPECT)/CT system in oral cavity squamous cell carcinoma Head Neck 2006 28 874 879 10.1002/hed.20434 16933311

[B21] De Cicco C Trifirò G Calabrese L Bruschini R Ferrari ME Travaini LL Fiorenza M Viale G Chiesa F Paganelli G Lymphatic mapping to tailor selective lymphadenectomy in cN0 tongue carcinoma: beyond the sentinel node concept Eur J Nucl Med Mol Imaging 2006 33 900 905 10.1007/s00259-006-0088-4 16604345

[B22] Daisne JF Blumhofer A Atlas-based automatic segmentation of head and neck organs at risk and nodal target volumes: a clinical validation Radiat Oncol 2013 8 154 10.1186/1748-717X-8-154 23803232 PMC3722083

[B23] Dirix P Abbeel S Vanstraelen B Hermans R Nuyts S Dysphagia after chemoradiotherapy for head-and-neck squamous cell carcinoma: dose-effect relationships for the swallowing structures Int J Radiat Oncol Biol Phys 2009 75 385 392 10.1016/j.ijrobp.2008.11.041 19553033

[B24] ICRU Prescribing, recording, and reporting photon-beam intensity-modulated radiation therapy (IMRT) J ICRU 2010 10 1 106

[B25] Vees H Steiner C Dipasquale G Chouiter A Zilli T Velazquez M Namy S Ratib O Buchegger F Miralbell R Target volume definition in high-risk prostate cancer patients using sentinel node SPECT/CT and 18 F-choline PET/CT Radiat Oncol 2012 7 134 10.1186/1748-717X-7-134 22873771 PMC3561224

[B26] Cheville AL Brinkmann DH Ward SB Durski J Laack NN Yan E Schomberg PJ Garces YI Suman VJ Petersen IA The addition of SPECT/CT lymphoscintigraphy to breast cancer radiation planning spares lymph nodes critical for arm drainage Int J Radiat Oncol Biol Phys 2013 85 971 977 10.1016/j.ijrobp.2012.08.025 23452455

